# Electrocardiographic parameters and prognosis of renal light chain amyloidosis

**DOI:** 10.1002/clc.23426

**Published:** 2020-07-28

**Authors:** Huixian Li, Ying Wang, Ping Lan, Liyi Xie, Yanhong Zhao, Wanhong Lu, Guoliang Li

**Affiliations:** ^1^ Department of Nephrology Kidney Hospital, The First Affiliated Hospital of Xi'an Jiaotong University Xi'an Shaanxi China; ^2^ Office of Health Care for Cadres the First Affiliated Hospital of Xi'an Jiaotong University Xi'an Shaanxi China; ^3^ Department of Cardiovascular Medicine the First Affiliated Hospital of Xi'an Jiaotong University Xi'an Shaanxi China; ^4^ Department of Network Information the First Affiliated Hospital of Xi'an Jiaotong University Xi'an Shaanxi China

**Keywords:** arrhythmia, cardiac involvement, electrocardiogram, mortality, renal light chain amyloidosis

## Abstract

**Background:**

Cardiac involvement frequently occurs in patients with renal light chain (AL) amyloidosis, which predisposes these patients to heart failure, arrhythmia, or infarction with poor prognosis.

**Hypothesis:**

Twelve‐lead electrocardiogram (ECG) parameters may be associated with prognosis in renal AL amyloidosis.

**Methods:**

A retrospective single‐center cohort study was performed. Biopsy‐proven renal AL amyloidosis patients from January 2014 to December 2018 at the First Affiliated Hospital of Xi'an Jiaotong University were enrolled. The baseline demographic information, laboratory tests, 12‐lead ECG parameters at the time of diagnosis were obtained from medical records. The endpoint was defined as the time to all‐cause death from baseline for all deceased patients and time to censor date (June 2019) for all other patients. Univariate and multivariate Cox proportional hazard models were conducted to identify the relationship between ECG parameters and all‐cause mortality.

**Results:**

A total of 69 patients with a mean age of 61.5 ± 11.4 years were enrolled in this study. The median PR interval and QTc interval were 160 (140, 186) and 417 ± 42 ms. The mean follow‐up duration was 15.9 ± 13.8 months. Multivariate Cox regression analysis showed that regardless of adjustment for age, gender and serum creatinine, PR interval (HR 1.022, 95% CI: 1.007‐1.038, *P* = .005), and QTc interval (HR 1.012, 95% CI: 1.004‐1.021, *P* = .004) were independently associated with all‐cause mortality.

**Conclusions:**

PR interval and QTc interval were independently associated with all‐cause mortality in renal AL amyloidosis patients. ECG parameters may provide prognostic potential of renal AL amyloidosis patients and promote the management of patients with renal AL amyloidosis.

## 1 INTRODUCTION

Light chain (AL) amyloidosis is a systemic disorder marked by the amyloid deposition of protein derived from immunoglobulin light chain fragments in multiple organs and it is a kind of rare diseases with a reported stable incidence of 9 to 14 cases per million person‐year in the United States.^1^, ^2^ The kidney is one of the most frequent sites of amyloid deposition. The prognosis of renal amyloidosis patients mainly depends on whether echocardiographic evaluation demonstrates infiltrative cardiomyopathy.^3^, ^4^ Therefore, assessment of cardiac burden is essential for estimating the prognosis and future treatment strategy.

Previous studies have suggested that left ventricular (LV) global longitudinal strain (GLS) measured from echocardiography was associated with prognosis of cardiac amyloidosis.^5^ However, the relationship between electrocardiogram (ECG) parameters and prognosis in renal amyloidosis has rarely been reported. We aimed to investigate the relationship between 12‐lead ECG parameters and all‐cause mortality in renal AL amyloidosis and evaluate the prognostic potential of renal AL amyloidosis patients.

## 2 MATERIALS AND METHODS

### 2.1 Participants

Patients who had biopsy‐proven renal AL amyloidosis confirmed by Congo red staining and immunohistochemistry from January 2014 to December 2018 at the First Affiliated Hospital of Xi'an Jiaotong University were, retrospectively, identified. Any patient with no follow‐up information, persistent atrial fibrillation, paced rhythm, or previous history of myocardial infarction was excluded. This study was approved by the Ethics committee of the First Affiliated Hospital of Xi'an Jiaotong University and informed consent was obtained from all subjects. All methods were carried out in accordance with the relevant guidelines and regulations according to the principles expressed in the Declaration of Helsinki.

### 2.2 Demographic and clinical information

**FIGURE 1:**
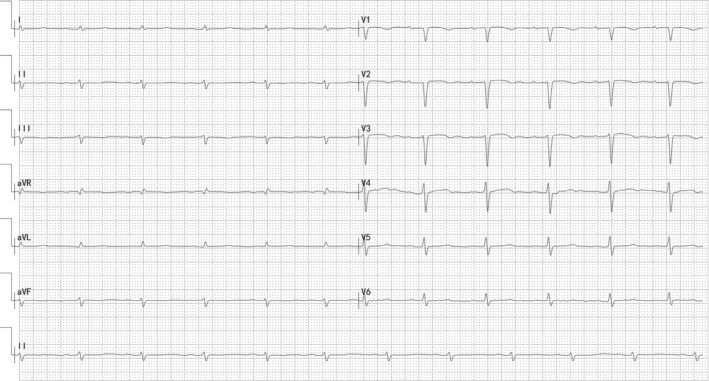
Electrocardiogram (ECG) from a 55‐year‐old male renal AL amyloidosis patient: PR interval 198 ms, QTc interval 476 ms, low limb voltage

**FIGURE 2:**
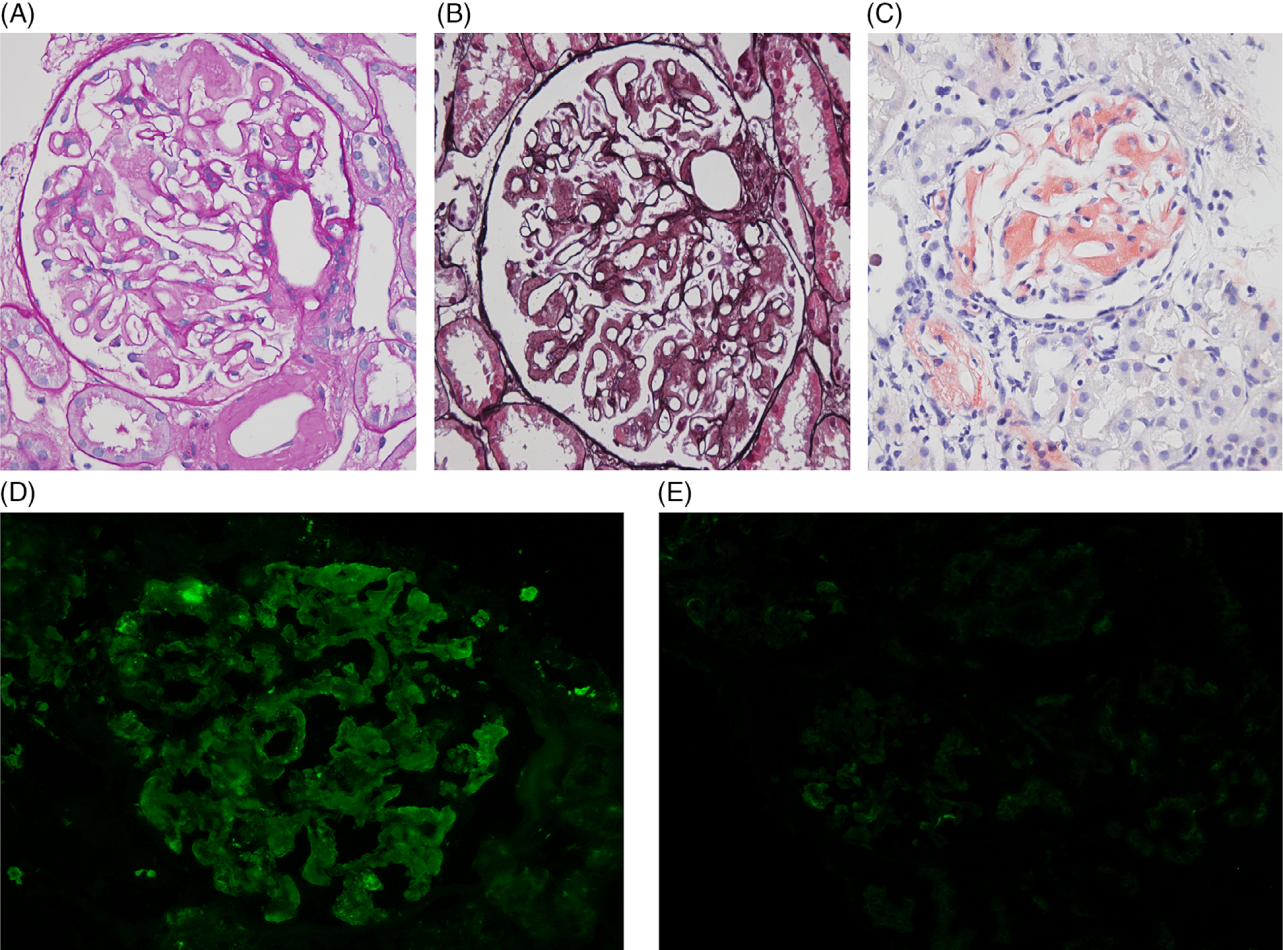
Diffuse glomerular deposition of amorphous material in the mesangium and the capillary loops, A, PAS staining. B, PASM and Masson staining. C, Congo red staining. Immunofluorescence microscopy. D, Positive for lambda light chains. E, Negative for Kappa light chains

The baseline demographic and clinical information which included hematologic laboratory tests, 12‐lead ECG and echocardiography at the time of diagnosis were obtained from medical records. The Chronic Kidney Disease Epidemiology Collaboration (CKD‐EPI) Study equation was used to calculate estimated glomerular filtration rate (eGFR). The endpoint was defined as the time to all‐cause death from baseline for all deceased patients and time to censor date (June 2019) for all other patients. Date of baseline was within 2 weeks of diagnosis. Occurrence and date of death, ongoing survival status were routinely monitored by regular visits or telephone calls. All ECG tracings were studied by two independent investigators, and a consensus was reached in cases where there was disagreement. All patients underwent echocardiography. LV end diastolic dimension, end systolic dimension, interventricular septal thickness, LV posterior wall thickness, and LV ejection fraction were measured. ECG and kidney biopsy results of a patient are shown in Figures 1 and 2.

### 2.3 Statistical analysis

Results were expressed as frequencies and percentages for categorical variables, mean ± SD for continuous variables, and median and interquartile range for nonparametric data. Differences in overall survival were assessed by log‐rank analysis and displayed by Kaplan‐Meier survival curves. Univariate and multivariate Cox proportional hazard model was conducted to adjust for potential confounders and identify independent predictors. All analyses were performed using SPSS version 20 (SPSS Inc., Chicago, Illinois). A *P* value <.05 was considered statistically significant.

## 3 RESULTS

### 3.1 Baseline characteristics

A total of 69 patients with a mean age of 61.5 ± 11.4 years were enrolled in our study. Table 1 shows the baseline demographic and clinical characteristics within 2 weeks of the kidney biopsy. The median time from onset to diagnosis was 6 (2, 12) months. The median eGFR was 99.0 ± 57.5 mL/min/1.73 m^2^.

**TABLE 1:** Baseline characteristics of all patients

No. patients	N = 69
Age (y)	61.5 ± 11.4
Male, n (%)	42 (60.9%)
Body mass index (kg/m^2^)	23.0 ± 2.3
Time to diagnosis (mo)	6 (2, 12)
Follow‐up duration (mo)	15.9 ± 13.8
Systolic blood pressure (mm Hg)	117.7 ± 20.8
Diastolic blood pressure (mm Hg)	74.8 ± 10.5
White blood cell (×10^9^/L)	7.3 (5.8, 8.7)
Hemoglobin (g/L)	127.0 ± 22.9
Platelet (×10^9^/L)	261.4 ± 107.3
Alanine aminotransferase (U/L)	24.7 ± 15.8
Cholesterol (mmol/L)	6.8 ± 3.3
Albumin (g/L)	25.5 ± 7.2
Globulin (g/L)	23.7 ± 8.2
Urea nitrogen (mmol/L)	6.7 (5.2, 9.9)
Creatinine (μmol/L)	69 (53, 111)
eGFR (mL/min/1.73 m^2^)	99.0 ± 57.5
Uric acid (mmol/L)	356.3 ± 120.6
24 h urine protein (g)	3.26 ± 2.47
Calcium (mmol/L)	2.1 ± 0.2
Lactate dehydrogenase (U/L)	254.5 ± 83.3
Troponin T (ng/mL)	0.049 ± 0.037
Serum β‐microglobulin (mg/L)	2572 (1905, 4286)
NT‐proBNP (ng/mL)	4013.1 ± 5786.8
Prothrombin time (s)	13.2 ± 1.1
Activated prothrombin time (s)	34.7 ± 6.7
*Electrocardiographic parameters*	
Rate	79 ± 15
PR interval (ms)	160 (140, 186)
Low voltage n (%)	9 (13)
RR interval (ms)	776.9 ± 181.0
QRS duration (ms)	82.7 ± 18.0
QT (ms)	377.2 ± 38.6
QTc (ms)	416.9 ± 42.2
*Echocardiographic parameters*	
LV end diastolic dimension (mm)	46.0 ± 6.3
LV end systolic dimension (mm)	28.5 ± 4.2
Interventricular septum (mm)	10.0 ± 2.3
LV posterior wall (mm)	8 (8, 10)
LV ejection fraction (%)	68.1 ± 6.4

*Note:* Low voltage: amplitude of QRS in each limb lead ≤0.5 mV or precordial lead ≤1 mV.

Abbreviations: eGFR, estimated glomerular filtration rate; LV, left ventricular; NT‐proBNP, N‐terminal prohormone of brain natriuretic peptide.

### 3.2 ECG parameters and patient survival

**FIGURE 3:**
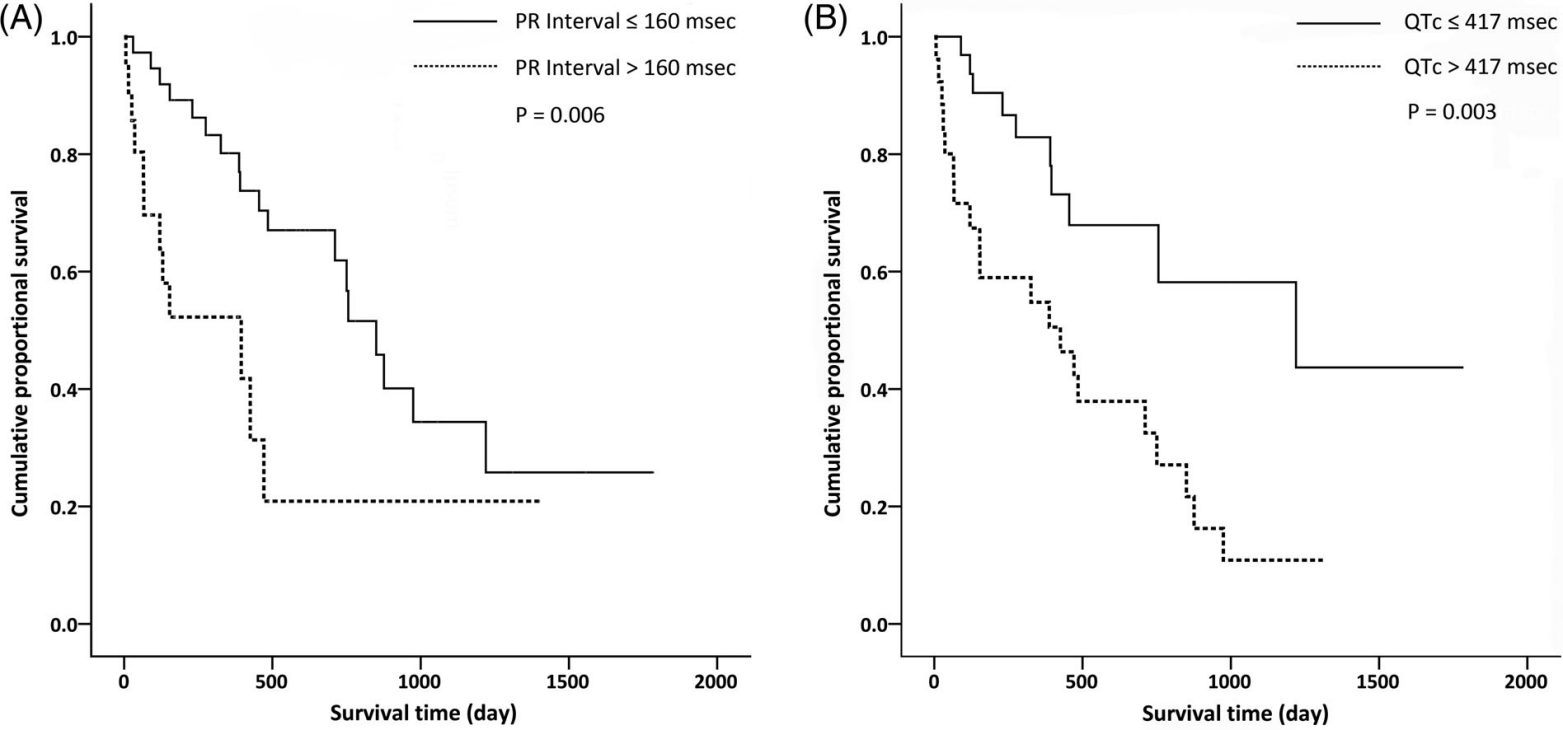
A, Kaplan‐Meier survival curves according to the presence of prolonged PR interval (>160 ms), log rank *P* = .006. B, Kaplan‐Meier survival curves according to the presence of prolonged QTc interval (>417 ms), log rank *P* = .003

**FIGURE 4:**
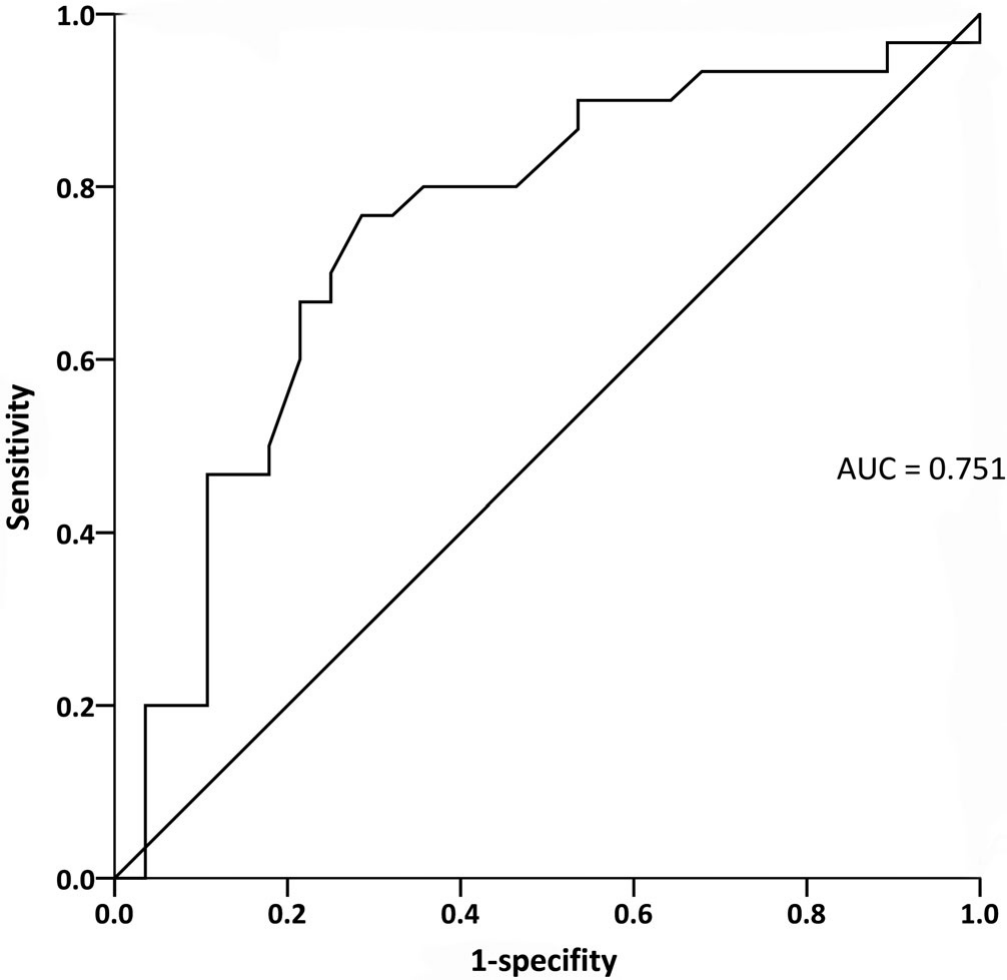
The QTc interval showed satisfactory predictive values for overall survival in renal AL amyloidosis (*P* = .001)

The average duration of follow‐up was 15.9 ± 13.8 months. During the follow‐up, 30 patients (43.5%) died. Univariate Cox analysis showed that hemoglobin, alanine aminotransferase, creatinine, prothrombin time, PR interval, and QTc interval were associated with patient survival. The results of multivariate Cox regression analysis for ECG parameters and other selected possible predictors of mortality in patients with AL amyloidosis showed that regardless of adjustment for age, gender and serum creatinine, PR interval, and QTc interval were independently associated with overall mortality (*P* = .005 and .004, respectively) (Table 2). We divided all patients into two groups according to the median level of PR interval (160 ms) and QTc (417 ms), the survival curves showed significant differences between two groups according to the Kaplan‐Meier plot (log rank test, *P* = .006 and .003, respectively) (Figure 3). The QTc interval showed satisfactory predictive values for overall survival in renal AL amyloidosis (Figure 4, cutoff value: 409.5 ms, sensitivity 76.6%, specificity 71.4%, AUC 0.751(95% CI: 0.620‐0.881, *P* = .001).

**TABLE 2:** Univariate and multivariate analyses for overall mortality

	Univariate			Multivariate		
	HR	HR (95% CI)	*P* value	HR	HR (95% CI)	*P* value
Age (y)	0.999	0.967‐1.033	.958	0.975	0.938‐1.012	.184
Male	0.842	0.403‐1.759	.647	0.485	0.190‐1.240	.131
Hemoglobin (g/L)	0.974	0.956‐0.992	.005			
Alanine aminotransferase (U/L)	1.021	1.001‐1.042	.038			
Albumin (g/L)	1.031	0.986‐1.079	.180			
Creatinine (μmol/L)	1.003	1.001‐1.005	.002	1.004	1.002‐1.007	.001
eGFR (mL/min/1.73 m^2^)	0.989	0.981‐0.997	.006			
Uric acid (mmol/L)	1.004	1.001‐1.007	.010			
24 h urine protein (g)	0.974	0.835‐1.136	.74			
Serum β‐microglobulin (mg/L)	1.000	1.000‐1.000	.044			
NT‐proBNP (ng/mL)	1.000	1.000‐1.000	.194			
Prothrombin time (s)	1.392	1.041‐1.861	.026			
PR interval (ms)	1.021	1.007‐1.036	.003	1.022	1.007‐1.038	.005
QTc (ms)	1.010	1.003‐1.017	.005	1.012	1.004‐1.021	.004
LV end diastolic dimension (mm)	1.011	0.951‐1.076	.722			
LV end systolic dimension (mm)	0.980	0.813‐1.181	.830			
Interventricular septum (mm)	1.299	0.953‐1.772	.098			
LV posterior wall (mm)	0.744	0.454‐1.219	.241			
LV ejection fraction (%)	1.018	0.918‐1.128	.741			

Abbreviations: eGFR, estimated glomerular filtration rate; LV, left ventricular; NT‐proBNP, N‐terminal prohormone of brain natriuretic peptide.

## 4 DISCUSSION

In this study, ECG parameters in biopsy‐proven renal AL amyloidosis were measured to evaluate the relationship between ECG parameters and overall mortality. PR interval and QTc interval were independently associated with all‐cause mortality.

The prognosis of AL amyloidosis varies considerably depending on the number and extent of organ involvement. Renal involvement which is common in AL amyloidosis most often presents as proteinuria and nephrotic syndrome, as well as renal insufficiency. Creatinine is used for definition and staging of chronic kidney disease and high serum creatinine levels are associated with increased risk of adverse outcomes, including death, cardiovascular events.^6^, ^7^ In our study, creatinine was independently associated with all‐cause mortality, consistent with previous findings in general populations. In addition, the presence and severity of cardiac involvement was the key prognostic factor that affected the outcome of AL amyloidosis.^8^, ^9^ The Mayo (2004) Stage and Revised Mayo Stage (2012) systems which incorporate N‐terminal prohormone of brain natriuretic peptide and cardiac troponin were useful for assessment of prognosis.^10^

In recent years, additional cardiac markers from electrocardiographic parameters (eg, QTc interval and QRS axis) and echocardiography (eg, LV GLS) were implied to be significant predictors of prognosis in AL amyloidosis patients with cardiac involvement.^5^, ^11^, ^12^ Actually, in patients with AL amyloidosis, isolated cardiac involvement was rare, occurring in isolation in only 4% of cases.^13^ In addition, patients with isolated cardiac disease do not manifest the diagnostic clues seen in multisystem disease, and may progress and die without a diagnosis. Therefore, it is crucial to facilitate constant surveillance of cardiac involvement in AL amyloidosis patients with a noncardiac site to improve overall survival. 12‐lead ECG is a less costly and noninvasive measure to estimate cardiac amyloidosis and may reflect the infiltrative nature of cardiac amyloidosis.

In patients with cardiac amyloidosis, low voltage in limb leads, poor R progression, and pseudoinfarction were the most common findings.^14^, ^15^, ^16^ Low voltage QRS, pseudoinfarction and fragmented QRS was associated with adverse outcomes in cardiac amyloidosis defined by either endomyocardial biopsy or echocardiographic evidence.^14^, ^17^, ^18^ However, in our study, the prevalence of low voltage in limb leads was far below that in other studies and did not correlate with poor prognosis. The possible explanation was the different study populations. Our study enrolled the patients confirmed by kidney biopsy and their cardiac involvement may not occur or be in early stage.

Patients with cardiac AL amyloidosis may have progressive conduction system disease, the severity of which may not be apparent from the surface ECG. Our study illustrated that prolonged PR interval even within the normal level may be associated with poor prognosis. PR interval includes time for atrial depolarization (the *P* wave) and conduction through the AV node and the His‐Purkinje system. Previous studies have shown that the sinus node may be most often involved pathologically and sinoatrial node fibrosis was a common morphologic abnormality of the conduction system in cardiac amyloidosis.^19^

The prolonged QTc interval indicated delayed cardiac repolarization and may reflect the infiltrative nature of the disease and amyloid burden.^16^ The possible explanations underlying prolonged QTc include electrolyte disturbances, myocardial ischemia, etc. For healthy, cardiac amyloidosis and some other diseases, QTc interval has been suggested to be associated with adverse outcomes.^20^, ^21^, ^22^, ^23^ Consistent with previous results, our study demonstrated that prolonged QTc interval was independently associated with overall mortality, even after adjusting for age, gender, creatinine, and PR interval. For a nephrologist, when AL amyloidosis was diagnosed by kidney biopsy, ECG parameters may provide additional information for comprehensive assessment to determine the severity of organ involvement and the prognosis. Further studies need to concentrate on 12‐lead ECG parameters in AL amyloidosis and confirm the incremental value for prognosis evaluation in multisystem AL amyloidosis with a positive biopsy from a noncardiac site.

There were several limitations of our study. First, this is a small, single‐center, observational cohort study and the sample size was not large enough for us to perform a better cox model to adjust for additional influencing variables. Secondly, renal pathological characteristics, therapeutic regimens, and responses were not included for evaluation of response status was not achievable due to limited data of patients in our cohorts. Thirdly, more sophisticated ECG and echocardiography parameters were not included in our study for a more comprehensive analysis.

In conclusion, cardiac involvement is the major determinant of overall survival for patients with AL amyloidosis. The diagnosis of cardiac amyloidosis is confirmed by demonstrating amyloid deposits on endomyocardial biopsy, which is invasive and difficult to perform, and patients in underdeveloped regions have less access to endomyocardial biopsy. Our study highlights the prognostic potential of 12‐lead ECG in renal AL amyloidosis and may promote the management of patients with renal AL amyloidosis.

## CONFLICT OF INTEREST

The authors declare no potential conflict of interests.

## AUTHOR CONTRIBUTIONS

Guoliang Li, Wanhong Lu, and Huixian Li designed the study. Huixian Li, Ying Wang, Ping Lan, and Yanhong Zhao collected the data. Huixian Li and Ying Wang analyzed the data. Huixian Li wrote the first draft of the paper. Liyi Xie, Guoliang Li, and Wanhong Lu critically revised the article. All authors read and met the ICMJE criteria for authorship.
